# The longitudinal impact of smartphone dependency on happiness among children: A latent growth modeling approach

**DOI:** 10.1371/journal.pone.0344529

**Published:** 2026-04-22

**Authors:** Minsol Kim, Hyorim Lee

**Affiliations:** 1 Center for Beautiful Aging, Kyungpook National University, Daegu, Republic of Korea; 2 Department of Home Economics Education, Teachers College, Kyungpook National University, Daegu, Republic of Korea; Tianjin University, CHINA

## Abstract

This study aimed to examine the longitudinal relationship between smartphone dependency and happiness among Korean children from fourth grade in elementary school through the first year of middle school (approximately ages 9–13), thereby providing an in-depth understanding of children’s emotional development in the digital environment. Longitudinal data from four waves (2018–2021) of the Korean Children and Youth Panel Survey (KCYPS), comprising a nationally representative cohort of fourth-grade students (N = 1,889), were analyzed. Descriptive statistics, repeated measures analysis of variance, correlation analysis, and latent growth modeling (LGM) were employed. Descriptive analyses indicated a gradual decline in children’s happiness over time, whereas smartphone dependency showed a modest increasing trend. However, latent growth modeling revealed that the slope of smartphone dependency was not statistically significant, and therefore a no-change model was adopted for this construct. Across all waves, smartphone dependency was consistently and negatively correlated with happiness. Furthermore, LGM results demonstrated that higher levels of smartphone dependency were associated with less favorable trajectories of children’s happiness over time. These findings underscore the importance of adopting a longitudinal and model-based perspective to clarify the relationship between smartphone use and children’s emotional well-being, and they offer empirical implications for educational and policy interventions aimed at supporting healthy development in the digital era.

## Introduction

Human beings universally pursue happiness—one of the most fundamental and inherent psychological motives of life. According to Maslow’s [1] hierarchy of needs, the highest level of self-actualization is ultimately connected to the pursuit of happiness, which is considered a core goal of human existence. Likewise, Diener et al. [2] emphasized that subjective well-being is a central motivator and determinant of quality of life; meanwhile, Ryan and Deci’s [3] self-determination theory conceptualized happiness as a natural outcome experienced when basic psychological needs—namely, autonomy, competence, and relatedness—are fulfilled, underscoring happiness as a core value of human life.

Happiness is a multifaceted concept encompassing satisfaction with life as a whole and the experience of positive emotional states. From the positive psychology perspective, happiness extends beyond momentary pleasure to include multiple dimensions, such as positive relationships, achievements, and life meaning [4]. This suggests that happiness is not limited to emotional experiences but is also closely associated with psychological functioning and life purpose. Previous studies have conceptualized happiness in terms of “subjective well-being”—defined as a psychological state comprising life satisfaction, the presence of positive affect, and the absence of negative affect [5].

Among children, happiness transcends emotional states and is closely associated with positive social interactions, the development of interpersonal skills, and self-esteem [4,6]. Happier children engage more actively in peer relationships, which, in turn, promotes the development of social skills and emotional self-concepts [7]. Furthermore, children’s subjective happiness—linked to demographic characteristics and personality traits—is known to contribute positively to both development and academic achievement [8]. Moreover, happiness experienced during childhood provides a critical foundation for life satisfaction and mental well-being during adulthood [2]. Indeed, as a relatively stable psychological characteristic, children’s happiness is closely related to their early emotional experiences and tends to remain stable over time [6,7]. Reportedly, happiness levels among South Korean children remain below the Organisation for Economic Co-operation and Development (OECD) average, largely owing to excessive academic pressure, a competitive education system, peer conflicts, and experiences of social exclusion [9].

As children transition from early childhood into the school years, they are required to not only establish social relationships with peers and teachers but also cope with growing academic demands. These developmental challenges may foster changes in happiness distinct from those observed during early childhood. Specifically, the transition from elementary to middle school is a critical period characterized by structural changes in the learning environment, the reorganization of peer groups, and shifting teacher expectations, all of which may undermine children’s sense of belonging and emotional stability. Such psychological burdens have been associated with decreased overall happiness [10]. Furthermore, as grade level increases, children’s happiness is increasingly influenced by various school-related factors, including academic burden, peer relations, and school climate; among these, school belonging has been identified as a crucial protective factor in maintaining emotional well-being, with children experiencing higher belongingness exhibiting greater emotional stability and sustained positive affect [11].

Along with these developmental and school-related influences, the growth of digital media use, particularly smartphones, has emerged as a significant environmental factor shaping children’s happiness. In South Korea, the smartphone penetration rate, which was only 3.8% in 2010, soared to 94.8% by 2023, making smartphones an indispensable medium for most of the population [12]. Among adolescents, ownership rates reached 98.2%, indicating that nearly all children and youth possess smartphones. This rapid diffusion has resulted in a substantial rise in smartphone use time among school-aged children and adolescent [13,14], alongside greater diversity in the types and number of applications accessed. Consequently, the digital environment’s expansion has progressively reshaped children’s daily lifestyles and emotional experiences, fueling growing scholarly interest in smartphone use’s psychological and emotional consequences.

Additionally, international research has documented similar patterns, reporting significant increases in smartphone ownership and use with increases in grade level. For instance, the German Life History Study, a child longitudinal study, revealed that smartphone use time significantly increased between ages 10 and 17, with a sharp rise observed in the proportion of adolescents using smartphones for over three hours per day after 2021 [15], Likewise, the UK Ofcom [16] report on children’s media use reported that 83% of children aged 12–15 own a smartphone, with 91% of those aged 14 and above spending over three hours daily on their devices. Similarly, Christakis et al.’s [17] study of U.S. adolescents found that smartphone use increased with grade level, with students spending an average of over 1.5 hours per day on smartphones even during school hours.

Smartphone use’s rising prevalence has fueled growing concerns regarding smartphone dependency, particularly among children and adolescents. A national survey on smartphone overdependence in South Korea [18] reported that 22.9% of lower elementary school students are at risk of smartphone overuse, the highest rate of increase across all age groups, reflecting the trend toward the early onset of digital addiction [19]. Smartphone dependency refers to a state of diminished self-control and persistent use despite negative consequences, encompassing the following three sub-dimensions: failure of control, salience, and problematic outcomes [20]. This dependency has been associated with various psychological and emotional issues, including decreased concentration, insufficient sleep, social isolation, depression, and anxiety [21].

Children’s self-regulation develops gradually, rendering them particularly vulnerable to smartphone dependency within a digital environment that, as demonstrated by Poulain et al. [15], is increasingly associated with reduced quality of life and emotional well-being in young populations. Recent studies have suggested that the relationship between smartphone use and happiness among children is not unidirectional, but rather depends on the quality and degree of dependency. While smartphone use undermines emotional stability and happiness, under certain conditions, it also fosters positive outcomes, including enhanced social connectedness and expanded access to information [22,23].

Given this context, educational policies worldwide are increasingly emphasizing restrictions on and pedagogical responses to children’s smartphone use. For instance, the Korean Ministry of Education [24] revised the Guidelines on Teachers’ Student Life Guidance to strictly prohibit smartphone use during class. However, in response, the National Human Rights Commission of Korea [19] criticized blanket confiscation policies for potentially infringing upon human rights, highlighting the need for balanced regulation and educational engagement. Similarly, the UK Department for Education officially announced a nationwide ban on smartphones in schools in 2024; meanwhile, Portugal has adopted restrictions to improve learning focus and emotional stability. Collectively, these measures reflect a growing policy-level recognition that smartphone use carries substantial implications for not only technology use but also children’s holistic development and well-being.

Children’s happiness is not a transient emotional state but a psychological resource that accumulates across developmental stages, functioning as a crucial indicator of quality of life [2]. Therefore, examining how happiness changes over time is central to understanding child development. This study adopted latent growth modeling (LGM)—a statistical method that is well-suited to longitudinal research, as it estimates both the direction and rate of intra-individual change in happiness over time. Moreover, LGM assesses how relatively stable external variables, such as smartphone dependency, influence both the initial level (intercept) and rate of change (slope) of happiness. This analytic framework advances beyond simple correlational approaches to demonstrate the structural relationships between digital experiences and child development, identifying both risk and protective factors in the digital age.

Accordingly, this study aims to examine smartphone dependency’s long-term role in children’s core developmental tasks—namely, self-regulation, peer relationship building, and emotional stability—during school years. By employing a longitudinal design and LGM, this study provides meaningful insights into how smartphone dependency influences changes in happiness over time, offering practical implications for educational and counseling interventions that enhance children’s emotional well-being in the digital era.

## Method

### Research questions and model

This study aimed to examine the longitudinal association between smartphone dependency and children’s happiness using a latent growth modeling approach. Specifically, the study addressed the following research questions:

**RQ1.** How do children’s smartphone dependency and happiness change over time across the study period?

**RQ2.** What are the initial level and rate of change in children’s happiness over time?

**RQ3.** How does smartphone dependency predict the initial level and longitudinal change trajectory of children’s happiness?

This study examined the structural relationship between children’s smartphone dependency and happiness from a longitudinal perspective using an LGM. Smartphone dependency was modeled as a fixed exogenous variable, as no significant changes were observed across the four measurement points. By contrast, children’s happiness was modeled as a linear growth trajectory, with both the intercept and slope estimated.

**Fig 1:**
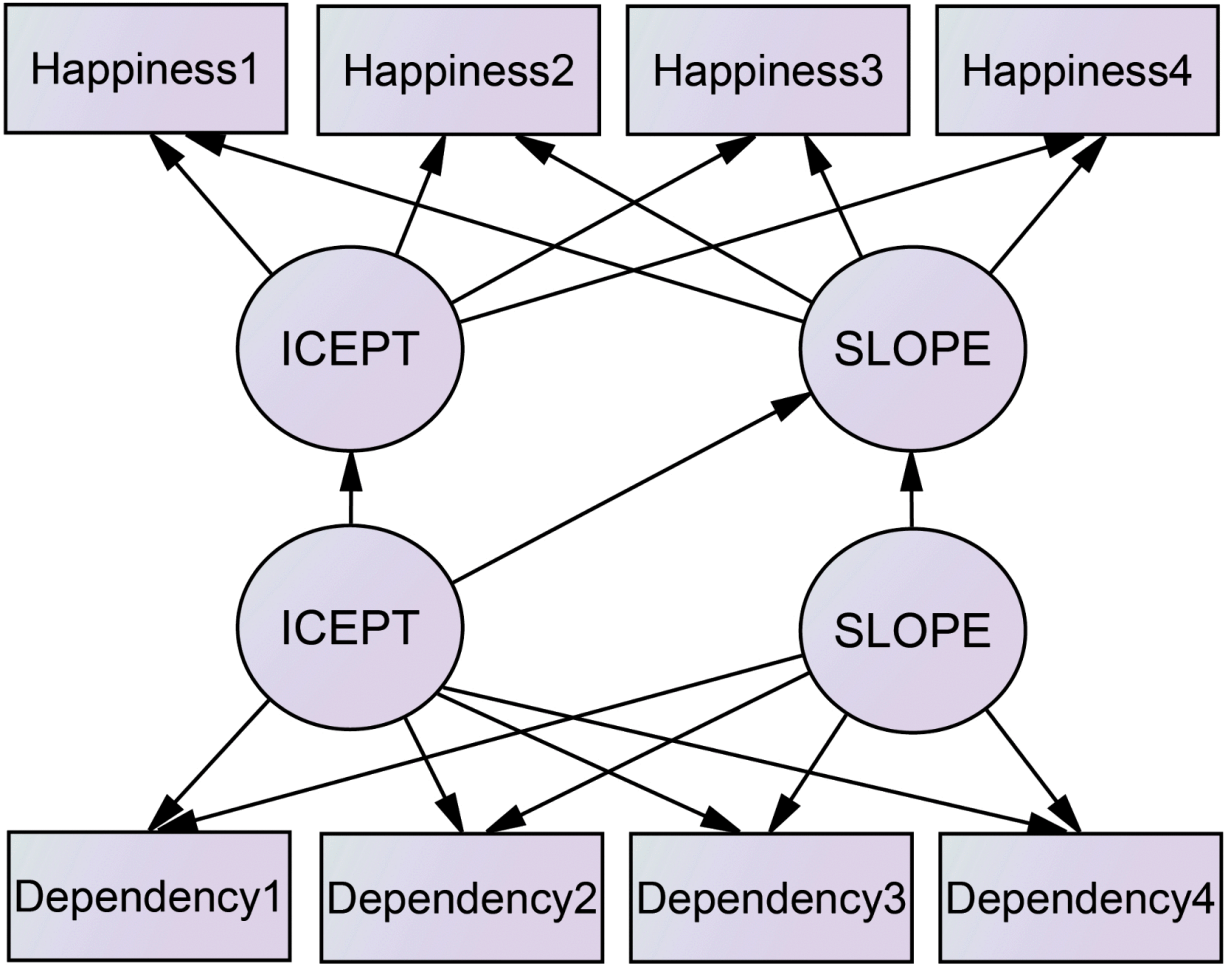
Conceptual framework of the study.

Accordingly, the research model was designed as a path model that structurally analyzes smartphone dependency’s influence on the initial level and rate of change in children’s happiness. Fig 1 illustrates the model.

### Participants

This study utilized data derived from the Korean Children and Youth Panel Survey 2018 [25], which examined children’s and adolescents’ personal growth and development, as well as their developmental trajectories over time. Building on the experiences of the original KCYPS launched in 2010, the KCYPS 2018 expanded its scope and target population; moreover, it has conducted annual follow-up surveys since 2018. For the baseline survey in 2018, samples were selected using a multistage stratified cluster sampling method, targeting fourth-grade elementary school students and first-year middle school students.

For this study, the cohort of fourth-grade elementary school students from the KCYPS 2018 was employed to analyze the longitudinal relationship between happiness and smartphone dependency. Fourth grade represents a transitional stage of middle childhood, during which various psychosocial characteristics—including emotional stability, self-concept, peer relationships, and academic stress—begin to develop more prominently [26,27]. Importantly, this period also coincides with a sharp increase in smartphone ownership and usage, though children’s capacity for self-regulation regarding digital media remains underdeveloped, posing heightened risks of overdependence.

According to the 2023 Smartphone Overdependence Survey conducted by the National Information Society Agency (NIA), the proportion of elementary school students at risk of smartphone overdependence increases significantly from the fourth grade onward, with problematic usage patterns becoming consolidated after the transition to middle school [20].

The KCYPS 2018 fourth-grade cohort tracked the same group of children annually for four years (2018–2021), which resulted in a robust longitudinal dataset that is well-suited for examining the long-term interplay between smartphone use and changes in happiness among children [25,28]. Accordingly, this study analyzed data derived from the first through fourth waves (2018–2021), focusing on participants who responded in all four waves and had no missing data on the key study variables. The final analytic sample comprised 1,889 children—specifically, 934 boys (49.4%) and 955 girls (50.6%)—reflecting a slightly higher proportion of girls.

### Measures

#### Happiness.

Children’s subjective happiness was measured using the Subjective Happiness Scale (SHS), which was originally developed by Lyubomirsky and Lepper [29]. For this study, the KCYPS research team translated and adapted the instrument for children. While the original SHS employs a 7-point Likert scale, the adapted version utilized herein was modified to a 4-point Likert scale to facilitate children’s comprehension, with item wording adjusted to be developmentally appropriate. Additionally, facial expression cards were presented to help children express their emotions more intuitively.

The scale comprised four items, with higher scores indicating greater happiness. A pilot study was conducted to refine item wording for children; the response format was modified into a 4-point scale (1 = not at all, 4 = very much). Data were collected through computer-assisted personal interviewing to enhance response accuracy. In this study, the reliability (Cronbach’s *α*) of the SHS was.759 (Wave 1),.775 (Wave 2),.708 (Wave 3), and.744 (Wave 4).

### Smartphone dependency

Children’s smartphone dependency was measured using the Smartphone Addiction Proneness Scale (SAPS) developed by Kim et al. (2014) [23]. The scale comprises 15 items across the following four subdomains: daily life disturbance (5 items), preoccupation with the virtual world (2 items), withdrawal (4 items), and tolerance (4 items).

Item examples include the following: “I have been scolded for frequently using my smartphone” (daily life disturbance); “Using my smartphone is more enjoyable than being with family or friends” (preoccupation with the virtual world); and “Spending a great deal of time on my smartphone has become a habit” (withdrawal). All items were rated on a 4-point Likert scale (1 = strongly disagree, 4 = strongly agree); reverse-coded items were recoded before analysis. Higher total scores indicate greater smartphone dependency. Each subdomain’s average scores were employed as observed variables in the analysis.

In the Korean Children and Youth Panel Survey (KCYPS), smartphone dependency items are provided as part of the secondary panel dataset and are administered using a recoded 4-point Likert-type scale for analytic consistency across waves. Although the original survey instrument was initially designed using a 7-point response format, the publicly released panel data apply a standardized recoding procedure prior to data distribution. The present study did not alter the original measurement structure but utilized the smartphone dependency variable as provided in the KCYPS dataset. This standardized response format facilitates longitudinal comparability across measurement occasions and reduces distributional sparsity in extreme response categories. Importantly, internal consistency reliability reported in the current analysis remained acceptable, indicating that the psychometric integrity of the scale was preserved within the panel data structure.

In this study, the reliability (Cronbach’s *α*) of the SAPS was.889 (Wave 1),.874 (Wave 2),.889 (Wave 3), and.852 (Wave 4).

### Data analysis

This study examined the longitudinal relationship between smartphone dependency and children’s happiness using an LGM. Before the main analyses, the reliability of the measurement instruments was assessed by computing Cronbach’s α coefficients to determine each subscale’s internal consistency. Descriptive statistics were conducted to identify the variables’ distributional characteristics and developmental trends across time points. Additionally, repeated measures analysis of variance (ANOVA) was performed to test the statistical significance of mean differences across waves.

For variables that exhibited significant differences across time, Bonferroni post-hoc tests were conducted to specify which time points differed significantly from one another. This procedure enabled a more accurate understanding of changes in smartphone dependency and happiness over time and at which time points significant differences emerged.

Further, Pearson correlation analyses were conducted to examine the basic relationships among the main study variables. Subsequently, LGM was employed to analyze the developmental trajectories and reciprocal associations between happiness and smartphone dependency. As a structural equation modeling technique, LGM simultaneously estimates individual change trajectories and inter-variable relationships over time. In this study, each variable’s trajectories were modeled as linear functions, and variance estimates for both the intercept and slope were utilized to capture individual differences in initial status and rate of change. This approach enabled the analysis of how initial levels and developmental rates differed across individuals, as well as the longitudinal interactions between happiness and smartphone dependency.

All analyses were conducted using the structural equation modeling software AMOS 18.0. Maximum likelihood estimation was applied for parameter estimation. Model fit was assessed using widely accepted indices that are relatively robust to sample size and consider model parsimony, including the root mean square error of approximation (RMSEA), Tucker–Lewis index (TLI), and comparative fit index (CFI). The proposed LGM’s adequacy was evaluated by comprehensively reviewing these fit indices.

### Ethics statement

This study used secondary data from the Korean Children and Youth Panel Survey (KCYPS). The survey protocol was reviewed and approved by the Institutional Review Board of the National Youth Policy Institute (NYPI), Republic of Korea. Written informed consent was obtained from parents or legal guardians of all participating children prior to the original data collection. The dataset used in the present study was fully anonymized and contained no personally identifiable information.

## Results

### Descriptive statistics and longitudinal changes in key variables

The longitudinal changes in happiness and smartphone dependency from fourth grade in elementary school to first grade in middle school were examined by conducting descriptive statistics and repeated measures ANOVA across four time points.

The mean happiness level was 3.37 (*SD* = 0.51) at the first measurement (Grade 4), indicating an overall high happiness level. However, a consistent decline was observed across subsequent waves: 3.22 (*SD* = 0.51), 3.13 (*SD* = 0.48), and 3.06 (*SD* = 0.48) at Waves 2, 3, and 4, respectively (Grade 7). The results of repeated measures ANOVA, with Greenhouse–Geisser correction applied, indicated that the mean differences across time were significant (*F*[2.84, 5341.45] = 212.37, *p* < .001, *η²* = .101). Further, polynomial contrasts demonstrated a significant linear decreasing trend (*F* = 484.37, *p* < .001, *η²* = .205).

By contrast, smartphone dependency exhibited the lowest mean score at the first measurement (*M* = 1.81, *SD* = 0.50), followed by a gradual increase: 1.98 (*SD* = 0.49), 2.12 (*SD* = 0.53), and 2.11 (*SD* = 0.46) at Waves 2, 3, and 4, respectively, indicating a general upward trend that stabilized in later waves. Moreover, the repeated measures ANOVA results, corrected using Greenhouse–Geisser, also indicated significant differences across time (*F*[2.91, 5477.84] = 321.16, *p* < .001, *η²* = .146). A significant linear increasing trend was likewise confirmed (*F* = 687.15, *p* < .001, *η²* = .267) (Table 1).

**Table 1:** Descriptive statistics and results of repeated measures analysis of variance for key variables.

Variable		*M*	SD	Skewness	Kurtosis	*F(df)*	*p*	*η²*
Dependent Variable	Happiness (Wave 1)	3.37	.51	−0.64	0.37	F(2.84, 5341.45) = 212.37	<.001	.101
	Happiness (Wave 2)	3.22	51	−0.22	0.04			
	Happiness (Wave 3)	3.13	.48	−0.05	0.47			
	Happiness (Wave 4)	3.06	.48	0.06	0.50			
Predictor Variable	Smartphone Dependency (Wave 1)	1.81	.50	0.53	0.17	F(2.91, 5477.84) = 321.16	<.001	.146
	Smartphone Dependency (Wave 2)	1.98	.49	0.22	−0.35			
	Smartphone Dependency (Wave 3)	2.12	.53	0.10	−0.54			
	Smartphone Dependency (Wave 4)	2.11	.46	0.08	−0.03			

Note. F values are reported based on Greenhouse–Geisser corrections.

### Post-hoc analysis of mean differences across time points

Considering the significant differences observed in the repeated measures ANOVA, Bonferroni-adjusted post-hoc tests were conducted to examine specific pairwise mean differences across time points.

For happiness, a gradual decline was observed from Wave 1 (Grade 4) to Wave 4 (Grade 7), with all pairwise mean differences being significant (*p* < .001). The largest difference was observed between Waves 1 and 4 (mean difference = –0.307), indicating a marked decline in happiness as grade level increased (Table 2).

**Table 2:** Results of bonferroni post-hoc analysis for mean differences in happiness.

Comparison		Mean Difference	*SD*	*p*	95% CI
Wave 1	Wave 2	−0.153*	0.013	.000	[-.187, -.118]
Wave 1	Wave 3	−0.235*	0.014	.000	[-.270, -.199]
Wave 1	Wave 4	−0.307*	0.014	.000	[-.344, -.270]
Wave 2	Wave 3	−0.082*	0.012	.000	[-.113, -.051]
Wave 2	Wave 4	−0.154*	0.013	.000	[-.188, -.121]
Wave 3	Wave 4	−0.072*	0.011	.000	[-.101, -.043]

*Note. p* < .05; CI = Confidence Interval.

For smartphone dependency, post-hoc results indicated a significant increase over time. Compared to Wave 1, the means for Waves 2, 3, and 4 were all significantly higher (*p* < .001). The largest mean difference was observed between Waves 1 and 3 (mean difference = –0.340), reflecting a notable rise in smartphone dependency. Although smartphone dependency continued to increase beyond Wave 2, the repeated-measures ANOVA post-hoc analysis indicated no significant difference between Waves 3 and 4 (p = 1.000), suggesting a plateau effect at the observed mean level from the third wave onward (Table 3).

**Table 3:** Results of bonferroni post-hoc analysis for mean differences in smartphone dependency.

Comparison		Mean Difference	*SD*	*p*	95% CI
Wave 1	Wave 2	−.196*	.012	.000	[-.228,-.163]
Wave 1	Wave 3	−.340*	.013	.000	[-.375,-.305]
Wave 1	Wave 4	−.333*	.013	.000	[-.368,-.298]
Wave 2	Wave 3	−.144*	.012	.000	[-.177,-.112]
Wave 2	Wave 4	−.134*	.013	.000	[-.170,-.104]
Wave 3	Wave 4	.007	.012	1.000	[-.023,.038]

Note.* p < .05; CI = Confidence Interval.

### Correlation between happiness and smartphone dependency

Overall, a significant negative correlation was found between happiness and smartphone dependency across all time points. Specifically, higher happiness levels were consistently associated with lower smartphone dependency levels.

At Wave 1, the correlation coefficient between happiness and smartphone dependency was –.31, indicating a significant negative association (*p* < .01). At Wave 2, the correlation coefficient was –.35, suggesting an even stronger negative correlation than at Wave 1 (*p* < .01), which implies that smartphone dependency’s detrimental effect on happiness became more pronounced at this stage. At Wave 3, a similar negative correlation was maintained (*r* = –.36, *p* < .01). At Wave 4, the correlation weakened slightly (*r* = –.26, *p* < .01), yet remained significant, demonstrating the persistence of a negative association between the two variables (Table 4).

**Table 4:** Correlations among key variables.

Variable	a	b	c	d	e	f	g	h
a. Happiness (Wave 1)	1.00							
b. Happiness (Wave 2)	.38^**^	1.00						
c. Happiness (Wave 3)	.31^**^	.47^**^	1.00					
d. Happiness (Wave 4)	.27^**^	.38^**^	.50^**^	1.00				
e. Smartphone Dependency (Wave 1)	−.31^**^	−.17^**^	−.11^**^	−.08^**^	1.00			
f. Smartphone Dependency (Wave 2)	−.20^**^	−.35^**^	−.17^**^	−.17^**^	.41^**^	1.00		
g. Smartphone Dependency (Wave 3)	−.18^**^	−.25^**^	−.36^**^	−.24^**^	.32^**^	.46^**^	1.00	
h. Smartphone Dependency (Wave 4)	−.13^**^	−.17^**^	−.18^**^	−.26^**^	.23^**^	.34^**^	.50^**^	1

**p < 0.01

### Latent growth model results: Trajectories of happiness and smartphone dependency

**Fig 2:**
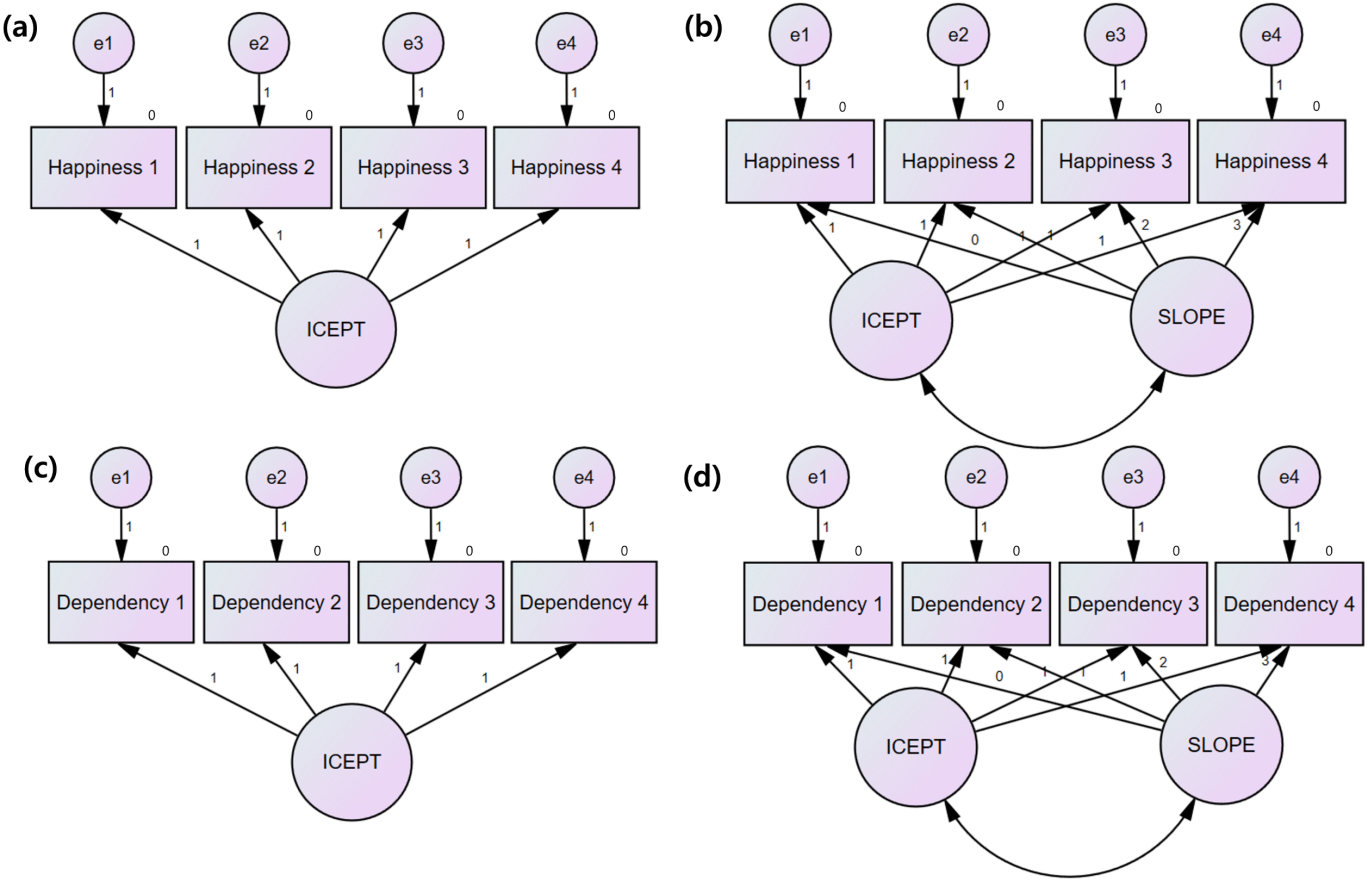
Comparison of Linear Growth and No-Change Models: (a) Happiness – No-Change Model; (b) Happiness – Linear Growth Model; (c) Smartphone Dependency – No-Change Model; (d) Smartphone Dependency – Linear Growth Model.

Fig 2 presents comparisons between no-change and linear growth models for happiness and smartphone dependency across four waves. Model fit indices indicated distinct longitudinal patterns for the two constructs.

For happiness, the no-change model showed inadequate fit (χ² [11] = 796.85, p < .001, TLI = .70, RMSEA = .20), whereas the linear growth model demonstrated substantially improved and acceptable fit (χ² [8] = 118.31, p < .001, TLI = .94, RMSEA = .09). Accordingly, the linear growth model was retained. Parameter estimates indicated a mean intercept of 3.35 and a significant negative mean slope (–0.30), reflecting a systematic decline in children’s happiness over time.

For smartphone dependency, both the no-change and linear growth models exhibited suboptimal fit according to conventional criteria. Although the linear growth model yielded modestly improved fit indices (χ² [8] = 241.41, p < .001, TLI = .88, RMSEA = .12) compared to the no-change model (χ² [11] = 934.92, p < .001, TLI = .65, RMSEA = .21), the improvement was insufficient to support a stable linear growth trajectory. Moreover, the estimated slope for smartphone dependency showed limited reliability, suggesting instability in the longitudinal change parameter. Given the marginal improvement in model fit and the instability of the slope estimate, the no-change model was selected to represent smartphone dependency in subsequent analyses.

In summary, happiness was best characterized by a linear decline over time, whereas smartphone dependency was modeled as a time-invariant construct within the latent growth framework, reflecting the absence of a well-fitting and stable growth trajectory (Table 5).

**Table 5:** Model fit indices and parameter estimates for no-change and linear growth models of happiness and smartphone dependency.

		*χ* ^*2*^	*df*	*p*	*TLI*	*RMSEA*	Intercept		Slope	
							Mean	Variance	Mean	Variance
Happiness	No-change	796.85	11	.000	0.70	0.20	3.20	.01^***^	–	–
	Linear	118.31	8	.000	0.94	0.09	3.35	.00	−0.30	0.14^***^
Smartphone Dependency	No-change	934.92	11	.000	0.65	0.21	2.01	.01^***^		
	Linear	241.41	8	.000	0.88	0.12	1.85	.01^***^	0.31	0.01^***^

### Model fit evaluation

The final model’s fit indices were as follows: *χ*²(10) = 133.18, *p* < .001, *TLI* = .92, *CFI* = .92, *RMSEA* = .08. Both the *TLI* and *CFI* values satisfied the conventional cutoff of.90 or above, indicating an adequately fitting model [30]. Additionally, the *RMSEA* was.081 with a 90% confidence interval of [.069–.093], which is within the upper bound of acceptable model fit [30]. Specifically, the RMSEA upper limit was below.10, while both *TLI* and *CFI* values reached.92, thereby satisfying the commonly accepted thresholds (≥.90). Collectively, these results suggest that the model demonstrated an acceptable level of fit.

Overall, the findings indicate that the structural model proposed herein adequately represents the data and offers a statistically valid framework for interpreting the relationship between smartphone dependency and changes in happiness among children (Table 6).

**Table 6:** Final Model’s Fit Indices.

	*χ* ^*2*^	*df*	*p*	*TLI*	*CFI*	*RMSEA*	90% CI
**Final Model**	133.181	10	<.001	.92	.92	.08	[.069,.093]

*Note.* TLI = Tucker–Lewis Index; CFI = Comparative Fit Index; RMSEA = Root Mean Square Error of Approximation; CI = Confidence Interval

### Path analysis of smartphone dependency’s effects on happiness among children

**Fig 3:**
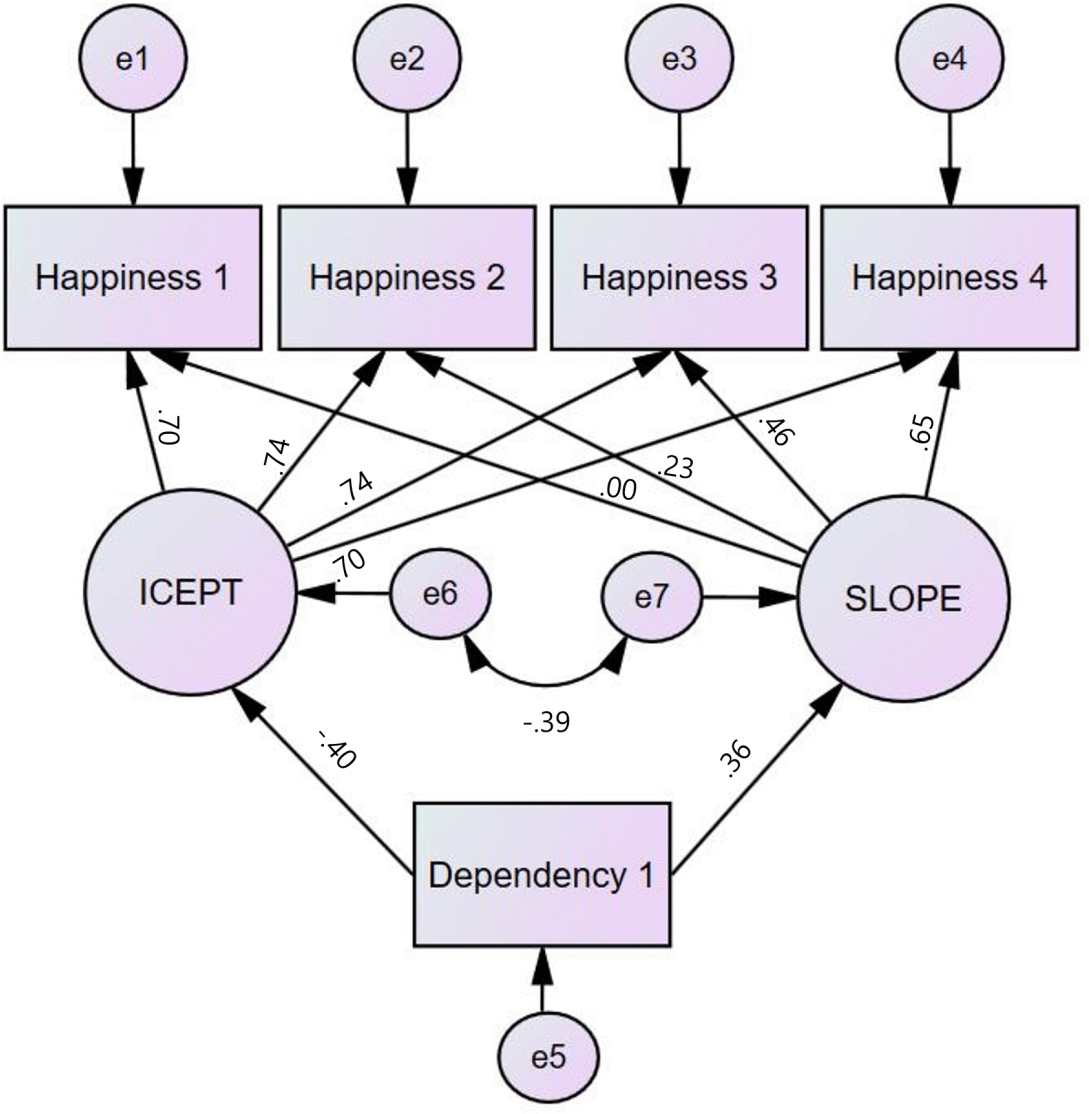
Final structural model.

Fig 3 illustrates the final structural model depicting the effects of smartphone dependency on the intercept and slope of children’s happiness. Smartphone dependency at Wave 1 significantly predicted both the initial level (intercept) and the rate of change (slope) in children’s happiness over time.

At Wave 1, smartphone dependency significantly negatively impacted the initial happiness level (*B* = –0.27, *β* = –0.40, *Z* = –13.86, *p* < .001), indicating that higher smartphone dependency levels were associated with significantly lower initial happiness levels.

Furthermore, smartphone dependency at Wave 1 significantly influenced the slope of happiness over time (*B* = 0.08, *β* = 0.36, *Z* = 8.84, *p* < .001). Specifically, higher smartphone dependency levels were associated with a greater rate of decline in happiness across time. These findings suggest that while smartphone dependency negatively impacts the baseline happiness level among children, it also shapes the trajectory of change, influencing the degree to which happiness diminishes over time (Table 7).

**Table 7:** Path coefficients of smartphone dependency’s effects on happiness among children.

Path	*B*	*β*	*S.E*	*Z*
Happiness Intercept ← Smartphone Dependency (Wave 1)	−0.29	−0.40	.02	−13.86^***^
Happiness Slope ← Smartphone Dependency (Wave 1)	0.08	0.36	.01	8.84^***^
Happiness (Wave 1) ← Happiness Intercept	1.00	0.70		
Happiness (Wave 1) ← Happiness Slope	0.00	0.00		
Happiness (Wave 2) ← Happiness Intercept	1.00	0.74		
Happiness (Wave 2) ← Happiness Slope	1.00	0.23		
Happiness (Wave 3) ← Happiness Intercept	1.00	0.74		
Happiness (Wave 3) ← Happiness Slope	2.00	0.46		
Happiness (Wave 4) ← Happiness Intercept	1.00	0.70		
Happiness (Wave 4) ← Happiness Slope	3.00	0.65		

***p < 0.001.

## Discussion and conclusion

This study aimed to deepen the current understanding of children’s developmental characteristics in the digital environment by analyzing the longitudinal relationship between smartphone dependency and happiness. Specifically, smartphone dependency was modeled as exhibiting no change, whereas happiness was modeled as demonstrating linear change; further, their trajectories were tracked over four years to empirically examine smartphone dependency’s long-term effects on happiness among children.

To explore the temporal dynamics between smartphone dependency and happiness among children, this study employed descriptive statistics, correlation analyses, and longitudinal analyses across four time points—from fourth grade in elementary school (Wave 1) to first grade in middle school (Wave 4). The repeated measures ANOVA results revealed that happiness was the highest at Wave 1 (fourth grade) but steadily declined through Waves 2 (fifth grade), 3 (sixth grade), and 4 (first grade in middle school). These changes were significant, and Bonferroni post-hoc analyses demonstrated that the mean differences between all the time points were significant.

Specifically, the mean difference between Waves 1 and 4 was the largest, suggesting that children’s emotional well-being markedly declines after middle childhood, indicating that happiness is not a static trait but rather fluctuates across developmental stages under the influence of various environmental and psychosocial factors.

These results can be interpreted in light of the psychological and environmental challenges that children encounter when transitioning from elementary to middle school. Upon entering middle school, they experience structural changes in their educational environment, including increased academic demands, subject-based teaching, and altered assessment systems, which may heighten academic stress and diminish self-esteem or self-efficacy [10]. Moreover, peer comparison and social competition become more salient, frequently undermining children’s sense of belonging and emotional stability [11]. Furthermore, the growing academic pressure before middle school entry—including intensified private tutoring, performance-oriented evaluations, and early involvement in entrance exam preparation—may limit children’s enjoyment and autonomy in non-academic activities, negatively influencing their emotional well-being [9].

Overall, these findings substantiate the perspective that happiness is not merely an internal emotional matter but a contextual outcome shaped by the complex interaction of developmental tasks and environmental demands. Strengthening school-based emotional support systems—for instance, by alleviating academic stress, enhancing peer relationships, and fostering a sense of belonging—is essential to sustaining children’s well-being during this critical transitional period.

By contrast, smartphone dependency was the lowest at Wave 1 (*M* = 1.81, *SD* = 0.50) and gradually increased through Waves 2 and 3, reaching 2.12 at Wave 3 (sixth grade) and stabilizing at 2.11 at Wave 4 (first grade in middle school). Repeated measures ANOVA demonstrated that this upward trend was significant (*F* = 687.15, *p* < .001, *η²* = .267). Bonferroni post-hoc analyses revealed that smartphone dependency increased significantly from Waves 1–3; however, no significant difference was found between Waves 3 and 4 (*p* = 1.000), which suggests that smartphone dependency stabilizes around the end of elementary school, highlighting this period as a strategic window for intervention.

This trend implies that smartphone use becomes habitual and entrenched in children’s daily routines by late elementary school, increasingly functioning as a means for social interaction, entertainment, and emotional regulation [16,21]. Considering children’s still-developing self-regulation abilities, problematic smartphone use may escalate into dependency, as reflected in rising scores over time. Such findings align with national and international surveys [16,18].

Further, the correlation analyses demonstrated consistently significant negative associations between happiness and smartphone dependency across all time points. Notably, initial smartphone dependency levels were negatively associated with both concurrent and later happiness levels, indicating that early patterns of problematic smartphone use exert lasting detrimental effects on emotional development among children.

The LGM analysis revealed different change patterns for the two variables. Happiness was best explained by a linear change model, with a significant negative slope (–0.30), indicating a consistent decline from Waves 1–4. By contrast, smartphone dependency was best represented by a no-change model, despite exhibiting a gradual increase in descriptive statistics. This suggests that while happiness is sensitive to environmental and developmental transitions, smartphone dependency reflects relatively stable and habitual patterns within the observed developmental period [21].

Although repeated-measures ANOVA indicated statistically significant mean-level increases in smartphone dependency over time, the latent growth modeling (LGM) results favored a no-change model for this construct. This apparent discrepancy reflects fundamental methodological differences between the two analytic approaches rather than contradictory findings. Repeated-measures ANOVA is primarily sensitive to average-level differences across time points and may detect statistically significant change even when the magnitude of change is small, particularly in large samples. In contrast, LGM simultaneously models both intraindividual change and interindividual variability in growth trajectories, providing a more stringent test of whether a stable and systematic developmental change pattern exists.

In the present study, although modest mean-level increases in smartphone dependency were observed across waves, the estimated growth trajectory did not demonstrate sufficient stability or model fit to support a linear change specification. Accordingly, smartphone dependency was modeled as a time-invariant construct within the LGM framework. Thus, the ANOVA and LGM findings should be interpreted as complementary rather than conflicting: the former captures small mean-level fluctuations, whereas the latter indicates the absence of a well-defined and stable growth trajectory when individual heterogeneity is taken into account.

Importantly, smartphone dependency at Wave 1 significantly predicted both the initial level (intercept) and rate of change (slope) in happiness. This finding underscores the criticality of early preventive interventions, ideally beginning before upper elementary school. Preventive approaches should extend beyond restricting smartphone use to fostering self-regulation, emotional expression, and opportunities for face-to-face interaction. Coordinated efforts between families and schools to integrate digital literacy with emotional regulation training are particularly vital before children reach fourth grade.

The divergence in change trajectories between happiness and smartphone dependency carries key methodological implications for future longitudinal research. Emotional and behavioral-environmental variables may follow distinct developmental patterns and, thus, should not be interpreted under a single trajectory model. For example, relatively stable variables, such as smartphone dependency, may be better modeled as fixed exogenous variables when analyzing their structural influence on emotional outcomes.

From a policy perspective, this study’s findings emphasize the importance of integrating preventive education into national strategies. Although regulatory approaches, such as restricting smartphone use during class, are necessary, they should be complemented by educational policies aimed at cultivating emotional regulation and responsible digital use. Specifically, the transitional stage from upper elementary to middle school should be prioritized for intervention—supported by school–family–community collaborations.

Despite its valuable contributions, this study has several limitations. First, both smartphone dependency and happiness were measured using self-report questionnaires, which may not fully capture actual behaviors. Second, the absence of control variables, such as gender, socioeconomic background, parental mediation, and peer relations, limits the findings’ generalizability. Third, the analysis was limited to a specific cohort of KCYPS data, warranting caution in broader application. Future research should incorporate longer-term longitudinal designs and consider both quantitative and qualitative aspects of smartphone use, including purpose, content, and social context. Advanced techniques, such as latent class growth modeling, latent profile analysis, and mixed-method approaches, could further refine understanding.

In conclusion, the present study demonstrated that children’s happiness exhibited a clear declining trajectory from late elementary school through early middle school, as evidenced by both mean-level analyses and latent growth modeling. The latent growth analysis further confirmed a significant negative slope, indicating a systematic developmental decline in happiness across the four-year period. These findings suggest that children’s emotional well-being is particularly sensitive to developmental and contextual transitions during this stage.

In contrast, smartphone dependency was best characterized by a relatively stable trajectory over time within the latent growth framework. Although mean-level increases were observed in descriptive and ANOVA-based analyses, the absence of a well-fitting and stable growth trajectory indicated that smartphone dependency functioned as a more habitual and time-invariant behavioral pattern once individual variability was taken into account. Importantly, early smartphone dependency significantly predicted both the initial level and subsequent change in happiness, highlighting the critical role of early preventive efforts. Taken together, these findings suggest that while children’s emotional well-being may undergo systematic developmental change, smartphone dependency reflects more entrenched behavioral tendencies that exert long-term influences on emotional development.
